# Protective Effects of Protocatechuic Acid on Seizure-Induced Neuronal Death

**DOI:** 10.3390/ijms19010187

**Published:** 2018-01-08

**Authors:** Song Hee Lee, Bo Young Choi, A Ra Kho, Jeong Hyun Jeong, Dae Ki Hong, Sang Hwon Lee, Sang Yup Lee, Min Woo Lee, Hong Ki Song, Hui Chul Choi, Sang Won Suh

**Affiliations:** 1 Department of Physiology, College of Medicine, Hallym University, Chuncheon 24252, Korea; sshlee@hallym.ac.kr (S.H.L.); bychoi@hallym.ac.kr (B.Y.C.); rnlduadkfk136@hallym.ac.kr (A.R.K.); jd1422@hanmail.net (J.H.J.); zxnm01220@gmail.com (D.K.H.); bluesea3616@naver.com (S.H.L.); 2 Faculty of Medical Sciences, Western University, London, ON N6A 5C1, Canada; sam8233157@gmail.com; 3 College of Medicine, Neurology, Hallym University, Chuncheon 24252, Korea; minwoo.lee.md@gmail.com (M.W.L.); hksong0@paran.com (H.K.S.); dohchi@naver.com (H.C.C.)

**Keywords:** epilepsy, pilocarpine, neuron death, protocatechuic acid, microglia, oxidative stress

## Abstract

Protocatechuic acid (PCA) is a type of phenolic acid found in green tea and has been shown to have potent antioxidant and anti-inflammatory properties. However, the effect of PCA on pilocarpine seizure-induced neuronal death in the hippocampus has not been evaluated. In the present study, we investigated the potential therapeutic effects of PCA on seizure-induced brain injury. Epileptic seizure was induced by intraperitoneal (i.p.) injection of pilocarpine (25 mg/kg) in adult male rats, and PCA (30 mg/kg) was injected into the intraperitoneal space for three consecutive days after the seizure. Neuronal injury and oxidative stress were evaluated three days after a seizure. To confirm whether PCA increases neuronal survival and reduced oxidative injury in the hippocampus, we performed Fluoro-Jade-B (FJB) staining to detect neuronal death and 4-hydroxynonenal (4HNE) staining to detect oxidative stress after the seizure. In the present study, we found that, compared to the seizure vehicle-treated group, PCA administration reduced neuronal death and oxidative stress in the hippocampus. To verify whether a decrease of neuronal death by PCA treatment was due to reduced glutathione (GSH) concentration, we measured glutathione with *N*-ethylmaleimide (GS-NEM) levels in hippocampal neurons. A seizure-induced reduction in the hippocampal neuronal GSH concentration was preserved by PCA treatment. We also examined whether microglia activation was affected by the PCA treatment after a seizure, using CD11b staining. Here, we found that seizure-induced microglia activation was significantly reduced by the PCA treatment. Therefore, the present study demonstrates that PCA deserves further investigation as a therapeutic agent for reducing hippocampal neuronal death after epileptic seizures.

## 1. Introduction

Epileptic seizures can lead to the development of several neurological disorders such as cognitive impairment [1,2,3]. The cause of seizures has not been clearly demonstrated. A traumatic brain injury or a stroke may cause a seizure by over-activation of the excitatory synaptic neurotransmission or by downregulation of the inhibitory neurotransmission [4]. Severe and prolonged seizures can cause neuronal death, which leads to the deterioration of cognitive functions [1].

The prognoses following severe and prolonged seizures have improved through several clinical approaches that include antibiotics and anticonvulsant drugs [5]. However, after a severe seizure, many survivors still display neuronal damage and cognitive decline. As demonstrated by several studies, oxidative stress is caused by an imbalance of reactive oxygen or nitrogen species (ROS/RNS). ROS usually plays an important role in the aging process of the brain and is indeed implicated in several neurodegenerative disorders, such as stroke, Parkinson’s disease, and Alzheimer’s disease. While the precise role that ROS plays in seizure-induced neuronal death remains to be defined, its general characteristic in such neuronal death is supported in part by the investigations of spontaneous seizures [6]: Free radical scavenging and reducing ROS production decreased neuronal death after seizures [7].

Glutathione (GSH) is a tripeptide, made of glutamate, cysteine, and glycine. It is known as a major cellular antioxidant and, by scavenging superoxide and reactive oxygen species in the brain, it plays an important role in reducing oxidative damage caused by ROS. It has also been shown that glutathione is important for the normal functioning of the brain [8]. GSH can be reproduced from glutathione disulfide (GSSG) via the glutathione reductase (GSR) enzyme. It has been shown to be effective against ROS and to defend neuronal toxic insults [9,10,11]. A deficiency of glutathione in the brain is associated with Parkinson’s disease, Alzheimer’s disease, seizures, and other neurodegenerative diseases [12,13].

Protocatechuic acid (PCA), a major metabolite of antioxidant polyphenols, is found naturally in green tea and in fruit. It is a dihydroxybenzoic acid, which is a phenolic acid. Protocatechuic acid is also found in various plants, such as cynamonowa, star anise, a medical rosemary, and melissa. It is a biologically active ingredient in some medicinal plants, such as Sudan Mallow (*Hibiscus sabdariffa* L.), Japanese ginkgo (*Ginkgo biloba* L.), and St. John’s wort (*Hypericum perforatum* L.). PCA has been widely recognized for its beneficial effects that include antioxidant, antibacterial, anti-ageing, anti-inflammatory, and neurological protective activities [14,15,16,17,18,19,20]. It has been proven that PCA reduces ROS generation. However, the efficacy of PCA treatment for seizure-induced neuronal death has not been verified.

In the present study, we used PCA (30 mg/kg, i.p.) [21] on a pilocarpine-induced animal seizure model. Here, we found that treatment with PCA after a seizure decreased oxidative injury, microglia activation, and neurodegeneration. We also found that the PCA treatment preserved neuronal GSH levels after a seizure. We therefore suggest that post-treatment with PCA could have a high therapeutic potential against seizure-induced neuronal death.

## 2. Results

### 2.1. Protocatechuic Acid (PCA) Treatment Reduces Seizure-Induced Hippocampal Neuronal Death

**Figure 1:**
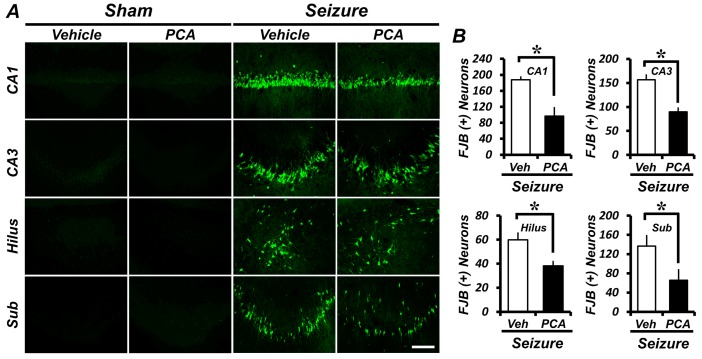
The protocatechuic acid (PCA) treatment reduces the number of degenerating neurons after a seizure. Three days of the PCA treatment reduces the number of seizure-induced degenerating neurons. Fluoro-Jade-B (FJB) staining was performed to visualize the degenerating neurons. (**A**) A series of photographs showing degenerating neurons in the control group and the PCA-treated group after a seizure. Scale bar = 100 μm. (**B**) The graph represents the number of FJB^+^ neurons in the hippocampus after a seizure. Data: mean ± standard error of the mean (SEM), *n* = 5–7 from each group. * *p* < 0.05. Veh = Vehicle; CA1 and CA3 = Cornu Ammonis 1 and 3 of the hippocampus area; Sub = Subiculum.

To assess whether PCA treatment exhibits neuroprotective effects, we evaluated the number of degenerating neurons by Fluoro-Jade-B (FJB) staining three days after a seizure. Degenerating neurons were observed in the CA1 and CA3 (Cornu Ammonis 1 and 3), hilus, and subiculum (Sub) areas (the region of the hippocampus) (Figure 1A). The PCA-treated group (30 mg/kg, i.p.) showed fewer FJB^+^ neurons when compared to the vehicle-treated control group. In the CA1, CA3, hilus, and subiculum areas, the degenerating neurons in the PCA-treated group were reduced by 48%, 43%, 36%, and 52%, respectively, when compared to the vehicle-treated group (Figure 1B).

### 2.2. PCA Treatment Preserves Neuronal Glutathione (GSH) Loss in Hippocampal Neurons

**Figure 2:**
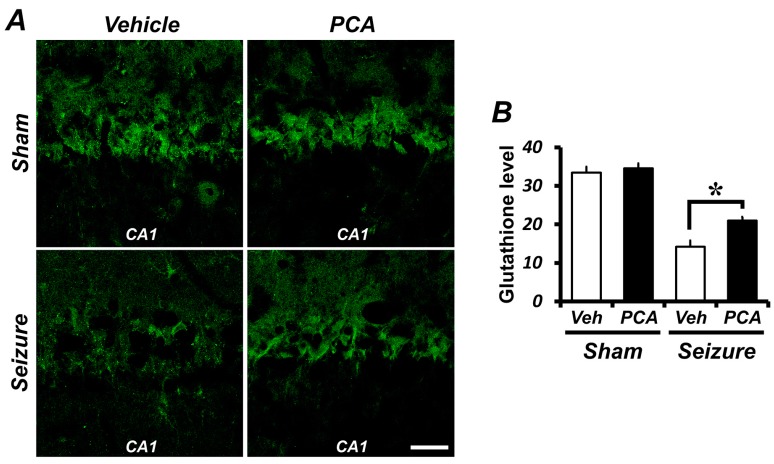
The PCA treatment reverses the glutathione (GSH) level in the hippocampal CA1 pyramidal neuron. (**A**) Confocal images showing glutathione with *N*-ethylmaleimide (GS-NEM, green) in CA1 pyramidal neurons of the hippocampus. The intensity of GS-NEM is decreased in the seizure-vehicle-treated group when compared to the sham-operated group. The GSH intensity is preserved in the seizure-PCA treated group when compared to the seizure-vehicle group. Scale bar = 100 μm. (**B**) The bar graph shows the level of neuronal GSH from the individual CA1 pyramidal neurons. Data: mean ± SEM, *n* = 3 from each group, * *p* < 0.05.

GSH levels were assessed in hippocampal pyramidal neurons by staining with glutathione with *N*-ethylmaleimide (GS-NEM) to confirm changes in GSH content. Compared to the sham group, the GSH level was significantly reduced in the seizure-induced group. The GSH concentrations in the PCA-treated group showed that, compared to the vehicle-treated group, there was a preservation of pyramidal neurons in CA1 (Figure 2). As shown in Figure 2B, in the hippocampal CA1 region, the GSH level decreased by 58% in the seizure-vehicle-treated group when compared to the sham-vehicle-treated group. On the other hand, the seizure group treated with PCA had a GSH level only 37% lower than the sham-vehicle-treated group. GSH levels were increased by 48% in the seizure PCA-treated group when compared to the seizure vehicle-treated group.

### 2.3. PCA Treatment Reduces Seizure-Induced Oxidative Injury in the Hippocampus

**Figure 3:**
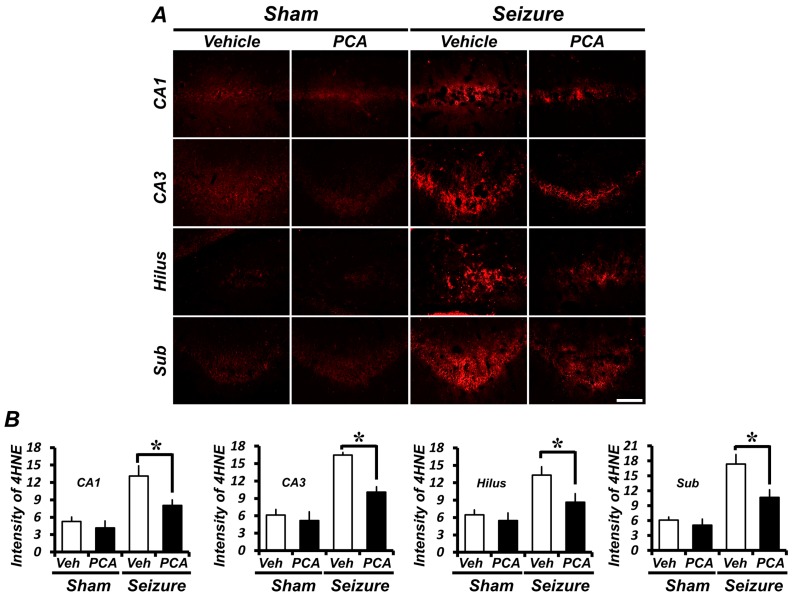
PCA reduces seizure-induced oxidative injury. Three days of treatment of PCA-reduced, seizure-induced oxidative injury. Oxidative stress was monitored by fluorescence microscopy after anti-4-hydroxynonenal (4HNE) immunostaining. (**A**) The intensity of 4HNE is increased in the seizure-vehicle group when compared to the sham-operated group. In the seizure-PCA treated group, the 4HNE intensity is lower than for the vehicle-treated group. Scale bar = 100 μm. (**B**) The bar graph shows the 4HNE fluorescence intensity in the hippocampus. Data: mean ± SEM, *n* = 5–7 from each group, * *p* < 0.05.

To detect oxidative damage after a seizure, we evaluated oxidative damage by using 4-hydroxynonenal (4HNE) staining. To determine whether oxidative stress was observed in hippocampal neurons, a 4HNE antibody was used to immunohistochemically stain rat-brain samples three days after a seizure. In the sham group, there was no difference in the 4HNE intensity of the hippocampus in both the saline injected vehicle and the PCA injected group. The intensity of 4HNE fluorescence was increased in the hippocampus of pilocarpine-induced seizure groups. However, in the PCA-injected group, the 4HNE fluorescence intensity was decreased after the seizure (Figure 3A). As estimated in Figure 3B, when compared to the sham-vehicle-treated group, the quantification of oxidative damage in the seizure-vehicle-treated group increased by 147%, 160%, 151%, and 170% for the CA1, CA3, hilus, and subiculum, respectively. On the other hand, the seizure PCA-treated group increased only by 51%, 60%, 59%, and 70% for the CA1, CA3, hilus, and subiculum, respectively, when compared with the sham-vehicle-treated group. The 4HNE intensity of the CA1, CA3, hilus, and subiculum were reduced, respectively, by 38%, 42%, 34%, and 40% when compared with the seizure PCA-treated group and the seizure vehicle-treated group (Figure 3B).

### 2.4. Seizure-Induced Hippocampal Neuronal Loss Is Reduced by PCA Treatment

**Figure 4:**
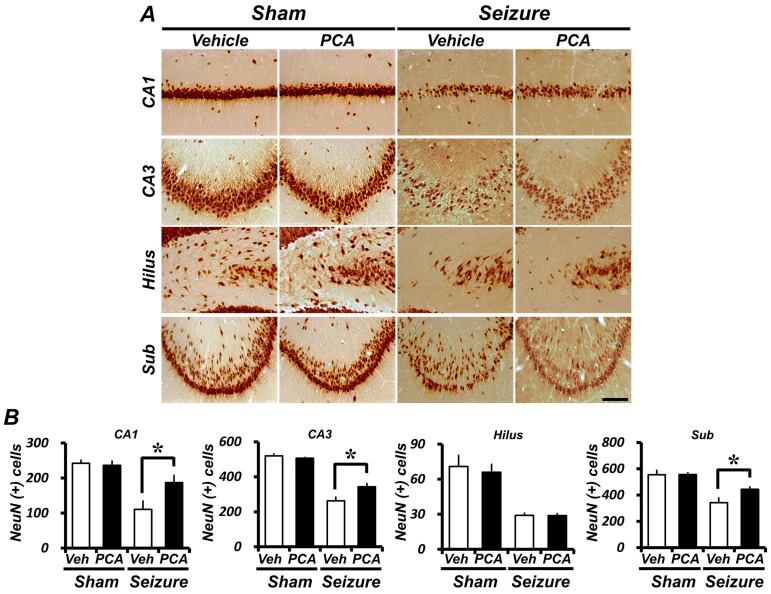
The PCA treatment reduces seizure-induced neuronal loss. Live neurons were detected by neuronal nuclear (NeuN) antigen immunohistochemical staining. Animals were either seizure-vehicle-treated or PCA-treated after a seizure. (**A**) is a representative image of the NeuN staining in the hippocampus and in the enlarged view of the CA1, CA3, hilus, and sub. Scale bar = 100 μm. (**B**) is a series of bar graphs indicating the number of NeuN^+^ neurons in the hippocampus after a seizure. When compared to the seizure-vehicle-treated group, the PCA-treated group had a higher number of NeuN^+^ neurons. Data: mean ± SEM, *n* = 5–7 from each group, * *p* < 0.05. The hilus is not statistically significant.

Immunohistochemical staining with an anti-neuronal nuclear (NeuN) antigen was performed to assay the survival of CA1, CA3, hilus, and subiculum neurons in the hippocampus. One week after the seizure, there were almost no NeuN-positive neurons in the CA1, CA3, or subiculum areas, when compared with the sham-vehicle group. However, a post-treatment with PCA increased the number of NeuN-positive cells in the CA1, CA3, and subiculum areas (Figure 4A). In the hippocampus, the number of live neurons in the vehicle-treated seizure group decreased by approximately 54%, 49%, and 38% in an enlarged view of the CA1, CA3, and subiculum when compared to the vehicle-treated sham group. However, the number of NeuN-positive neurons increased significantly in the seizure PCA-treated group. In the PCA-treated seizure group located in the hippocampus, the number of live neurons decreased only by approximately 22%, 34%, and 20% in the CA1, CA3, and subiculum, respectively, when compared with the vehicle-treated sham group. In the seizure PCA-treated group, NeuN-positive neurons increased by 58%, 28%, and 31% when compared to the seizure-vehicle-treated group (Figure 4B).

### 2.5. Seizure-Induced Microglia Activation Was Reduced by PCA

**Figure 5:**
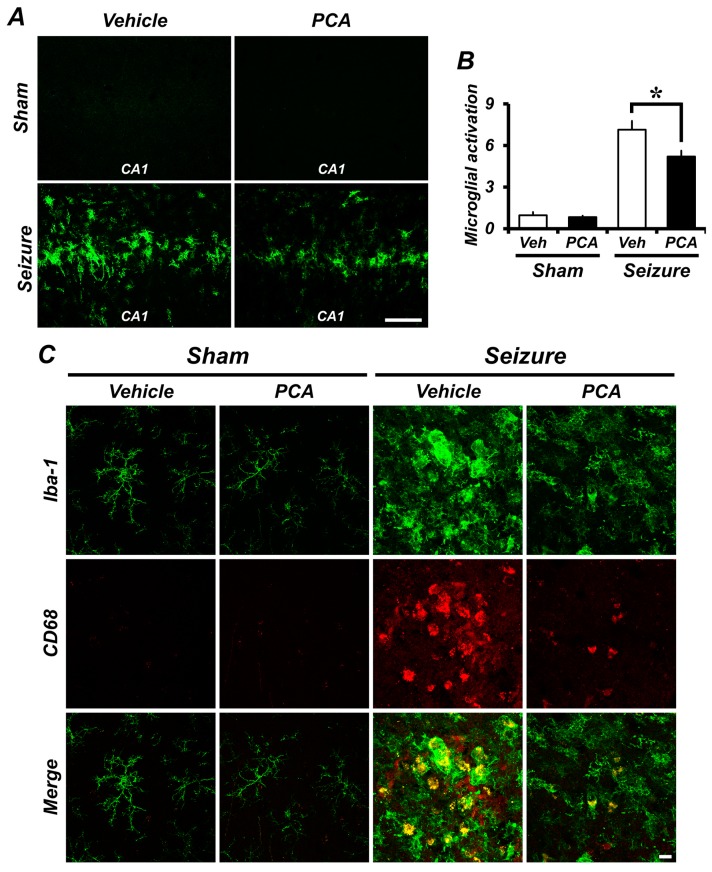
PCA treatment reduces microglia activation. Seizure-induced microglia activation was reduced by PCA treatment. Microglia activation was evaluated by CD11b staining. (**A**) shows microglia activation in CA1 of the hippocampus. One week after the seizure, microglia activation had occurred in the seizure group. In the CA1 area of the hippocampus, the PCA treatment reduced microglia activation when compared to the vehicle-treated group. Scale bar = 100 μm. (**B**) shows quantitated microglia activation in the hippocampus. (**C**) Iba1 and CD68 immunoreactive cells were expressed in the hippocampus. Iba1 and CD68 activation had occurred in the seizure group. The activities of Iba1 and CD68 were decreased in the PCA-treated group. Data: mean ± SEM, *n* = 5–7 from each group, * *p* < 0.05.

Seizures promote microglial activation via an indirect cell-death signaling pathway. Seizure-induced microglia activation is characterized by a gradual change in morphology, a proliferation, and a migration to the damaged region [22,23,24]. Staining of microglia was performed in activated microglia using an antibody against CD11b. We assessed the microglial activation among four groups. When compared to the sham-vehicle-treated group, microglial activation increased most dramatically in the seizure-induced group. Activated CD11b^+^ cells in the seizure PCA-treated group decreased by 27% when compared to the seizure vehicle-treated group. In addition, we also found that ionized calcium binding adaptor molecule 1 (Iba1) and cluster of differentiation 68 (CD68) is expressed on activated microglia. Seizures induced an increased number of Iba1/CD68^+^ cells. However, in the seizure PCA-treated group, the number of Iba1/CD68^+^ cells was less than that in the seizure-vehicle-treated group. The data therefore reveal that PCA negatively regulates microglial activation (Figure 5).

## 3. Discussion

In this study, we evaluated the effects of PCA on neuronal death after a pilocarpine-induced seizure. The neuroprotective effects of PCA have already been tested in ischemia and Parkinson’s disease models [25,26]. However, the effects of treating pilocarpine-induced seizures with PCA have not previously been evaluated. The present study tested whether hippocampal neuron death following a pilocarpine-induced seizure is reduced through PCA administration. We found that the PCA treatment showed neuroprotective effects through the reduction of ROS production and microglia activation, and through GSH restoration.

To evaluate whether the PCA treatment has neuroprotective effects, we analyzed degenerating neurons, superoxide production, GSH concentration, and microglia activation after a seizure. We observed an increase in the number of neurons in the hippocampus stained with FJB. Three days after the seizure, an analysis of the number of degenerating neurons showed that there was a significant decrease for the PCA-treated groups when compared to the vehicle-treated groups.

Oxidative damage has been implicated in promoting a variety of cell-death signal pathways [27] and is thought to play an important role as a causative factor of degenerative neuronal diseases [28,29]. The seizure-induced reduction of the GSH level and the increase of superoxide production in the hippocampus have been recognized as neuronal death mechanisms. Oxidative stress occurs when the free radical production exceeds the body’s neutralizing capacity. The main function of GSH is to buffer free radical activity before it leads to oxidative stress. The present study also found that neuronal GSH decreased and that ROS production increased three days after the seizure. The PCA administration reduced oxidative stress in seizure-induced rats. This finding further confirms and expands the idea that PCA reduces oxidative stress. We therefore believe that PCA treatment after a seizure is useful for reducing seizure-induced neuronal death. We found that a decrease in the neuronal GSH concentration led to the enhanced production of superoxide. Together, these results suggest that neuronal GSH levels in neurons contribute to a neuron’s ability to reduce superoxide and to suppress oxidative injury after seizure-induced neuronal death [30].

To verify whether a decrease in neuronal death by PCA treatment was the result of reduced oxidative injury, we measured GSH levels in the hippocampal neurons. Glutathione is an important cellular antioxidant that protects cells from being damaged by oxidative products. Antioxidants such as reduced GSH offset the deleterious effects of ROS. The recovery of neuronal GSH via a PCA administration can therefore decrease overall ROS production, eventually protecting against neuronal death after a seizure. Our present study, as well as studies performed by other laboratories, have shown that seizures induced a drop of GSH levels in the hippocampus [31]. This seizure-induced reduction of GSH concentrations in hippocampal neurons was preserved via PCA treatment. Thus, our study suggests that the recovery of GSH levels resulting from PCA treatment may support a neuroprotective effect following a seizure.

In order to determine whether the enhancement of GSH levels and the reduction of oxidative damage had a truly neuroprotective effect, we performed NeuN staining one week after the seizure. At that point, the PCA-treated group showed more live neurons in the hippocampus than the vehicle-treated group did. This result demonstrates that the one-week PCA treatment reduced neuronal death, eventually increasing the number of surviving neurons after the seizure.

Microglia are resident immune cells of the central nervous system (CNS) that play a crucial role in the detection and elimination of foreign molecules entering the brain. Microglia are a functional part of the CNS adapted to protecting fundamentally vulnerable cells, especially nerve cells, from damage [32]. Damaged neurons can induce microglia activation, but excessive microglia activation also has deleterious effects on neuronal survival. In several brain injuries, including seizures, the activation of microglia produces proinflammatory factors and ROS [33]. Activated microglia are associated with ROS production, which may induce or exacerbate neurotoxicity via oxidative stress to neurons [34,35]. We therefore examined whether microglia activation is affected by PCA treatment after a seizure. Here, we found that microglia activation was significantly reduced through PCA treatment. These results suggest that PCA confers neuroprotective effects through downregulation of the inflammatory response.

Disturbance of the antioxidant system increases the production of ROS in epilepsy patients [36]. Thus, several studies have suggested that treatment with antioxidants could decrease neurodegeneration, epileptogenesis, and milder cognitive deterioration after epilepsy [37,38,39,40,41,42]. Since phenolic acids are powerful antioxidants and have been known as antibacterial, antiviral, and anti-inflammatory actions, PCA, one of the main derivatives, may have neuroprotective effects after epilepsy.

## 4. Materials and Methods

### 4.1. Ethics Statement

This study was conducted in accordance with the guidelines in the Laboratory Animal Care and Use Guide, published by the National Institutes of Health (NIH). The animal studies were consistent with the requirements of the Hallym University Animal Care Committee (Project identification code # Hallym 2016–19, Approved at 30 June 2016). All attempts were made to minimize the suffering of the sacrificed animals with, for instance, isoflurane anesthesia.

### 4.2. Experimental Animals

Sprague–Dawley male rats (250–300 g, Daehanbiolink (DBL), Chungcheongbuk-do, Korea), aged 8 weeks, were used. Subjects were maintained at a constant room temperature (22 ± 2 °C) and humidity (55 ± 5%) and were housed one per cage, and room lights were automatically turned on and off on a 12 h cycle (on at 6:00 a.m. and off at 6:00 p.m.). The experimental animals were divided into four groups as follows: sham-vehicle *n* = 5, sham-PCA *n* = 5, seizure-vehicle *n* = 6, seizure-PCA *n* = 7.

### 4.3. Seizure Induction

Lithium chloride (LiCl, 127 mg/kg, i.p., Sigma-Aldrich, St. Louis, MO, USA) was administered to the animals 19 h before the injection of pilocarpine (25 mg/kg, i.p., Sigma-Aldrich, St. Louis, MO, USA) to cause status epilepticus (SE) [43]. Scopolamine (2 mg/kg, i.p., Sigma-Aldrich, St. Louis, MO, USA) was injected 30 min before the injection of pilocarpine. SE has been reported to occur 20–30 min after pilocarpine injection [44]. Animals were housed one per cage for individual observation. According to Racine’s method, animals exhibited up to five symptoms: (1) mouth and facial movements, (2) head nodding, (3) forelimb clonus, (4) rearing with forelimb clonus, and (5) rearing and falling with forelimb clonus. When the fifth symptom occurred, we judged that the seizure had occurred completely [45]. Two hours after the start of SE, diazepam (Valium, 10 mg/kg, i.p., Hoffman la Roche, Neuilly sur-Seine) was administered. Even after the diazepam treatment, repeated seizure behavior was observed in some animals [46]. When recurrent severe seizure was observed, additional diazepam was injected (2 mg/kg, i.p.) to stop the remaining seizure activity [47] (Figure S1).

### 4.4. Protocatechuic Acid (PCA) Injection

After the pilocarpine-induced seizure, animals were injected with PCA (30 mg/kg, i.p.) once per day for either 3 days or for 1 week. PCA was injected 2 h after the seizure onset, and a second injection was performed 24 h after the seizure. Alternatively, the PCA-vehicle group was treated with 0.9% normal saline. Seizure animals were sacrificed after 3 days and 1 week to evaluate neuronal death, oxidative injury, and microglial activation.

### 4.5. Brain Sample Preparation

Animals were sacrificed 3 days and 1 week after the seizures. Animals were deeply anesthetized via urethane injection (1.5 g/kg, i.p.). Anesthetized animals were perfused with 0.9% normal saline, then with 4% paraformaldehyde. Brain samples were post-fixed in the same fixative for 1 h, after which the brains were removed submersed in 30% sucrose overnight. After the brains sank to the bottom, usually 1~2 days after brain samples were harvested, the entire brain was frozen in dry ice. The entire brains were cut with a cryostat to a thickness of 30 μm and placed in a storage solution until the histological evaluation.

### 4.6. Detection of Live Neurons

To evaluate whether PCA has potential neuroprotective effects following a pilocarpine-induced seizure, a NeuN immunohistochemical stain was performed via brain section. A monoclonal anti mouse-NeuN antibody, at 4 °C (diluted 1:500, Billerica, Millipore, MA, USA), was employed overnight as the primary antibody. Brain sections were incubated with the biotinylated anti-mouse immunoglobulin G (IgG) (Vector, Burlingame, CA, USA) and with ABC compounds (Vector, Burlingame, CA, USA) diluted 1:250 in the identical compound of the primary antiserum. Between incubations, each section was washed three times for 10 min with phosphate-buffered saline (PBS). The immune response was developed with 3,3-diaminobenzidine (DAB, Sigma-Aldrich, St. Louis, MO, USA), and the sections were mounted on slides. The immune response was observed under an Olympus IX70 inverted microscope (Olympus, Tokyo, Japan) [48].

### 4.7. Detection of Degenerating Neurons

FJB staining revealed degenerating neurons in brain slices obtained 3 days after the seizures. [49,50]. To investigate the neuroprotective effect of PCA, 5.0 mm posterior-to-bregma sections were made, out of which every third was collected. FJB-positive neurons were counted in both hemispheric hippocampal CA1, CA3, hilus, and subiculum regions. The average number of FJB^+^ neurons in each region was used for statistical analyses.

### 4.8. Detection of Microglia Activation Macrohage Activation

Immunostaining was performed on coronal brain-tissue sections with a thickness of 30 μm. Five sections were analyzed from each brain. After washing them with 1 mM PBS, mouse antibodies against rat CD11b (diluted 1:500, AbD Serotec, Oxford, UK), rabbit anti-Iba1 (diluted 1:500, Abcam, Cambridge, UK), and mouse anti-CD68 (diluted 1:100, Biorad, Berkeley, CA, USA) were stained immunohistochemically with blocking buffer (10% goat serum and 0.1% Triton X-100 in 1 mM PBS) and incubated overnight in a 4 °C incubator. After washing, the sections were treated with a secondary antibody in a 1:250 dilution of Alexa Fluor 594-conjugated donkey anti-mouse IgG (Invitrogen, Grand Island, NY, USA) for 2 h at room temperature. Microglial activation was measured by a randomly blinded observer. Five sections from each sample were evaluated for scoring. The number, intensity, and morphology of CD11b immunoreactive cells were used as the baseline for microglial activation. Consequently, the overall score combined three scores (CD11b-immunoreactive cell number, morphology, and intensity) according to the previously reported categories, ranging from 0 to 9 [22,23].

### 4.9. Detection of GSH levels

To evaluate the glutathione level, we incubated rat brains in a 4 °C shaker for 4 h with a solution containing 10 mM *N*-ethylmaleimide (Nem, Sigma-Aldrich, St. Louis, MO, USA). The sample was then washed with 1 mM PBS and then directed against the GS-NEM (diluted 1:100, Millipore, Billerica, MA, USA). The brain sections were diluted with Alexa Fluor 488-conjugated goat anti-mouse IgG secondary antibody at 1:400 (Molecular Probes, Invitrogen) and treated in the dark for 2 h at room temperature. The selected hippocampal sections were measured for GSH levels using Image-J (NCBI, Bethesda, MD, USA).

### 4.10. Detection of Oxidative Injury

The detection of oxidative damage in the hippocampus was assessed via 4HNE (4-hydroxy-2-nonenal) staining for a lipid peroxidation product. 4HNE (Alpha Diagnostic International Inc., San Antonio, TX, USA) antibodies immunostaining was performed as previously described [51]. In the polyclonal rabbit anti-HNE antiserum compound (diluted 1:500, Alpha Diagnostic International Inc., San Antonio, TX, USA) tissues were incubated overnight at 4 °C in PBS containing 0.3% Triton X-100. Sections were washed in PBS three times for 10 min and incubated in a compound of Alexa Fluor 594-conjugated goat anti-rabbit IgG secondary antibody (Invitrogen, Grand Island, NY, USA) at a dilution of 1:250 for 2 h at room temperature (RT). After incubation, the sections were washed three times for 10 min in PBS and mounted on gelatin-coated slides.

### 4.11. Data Analysis

We compared each experimental group using repeated measures analysis of variance (ANOVA) and the Bonferroni post-hoc test. Data were presented as mean ± S.E.M., and differences were considered significant when *p* < 0.05.

## 5. Conclusions

In the present study, we firstly tested whether this antioxidant, PCA, has any therapeutic potential on seizure-induced neuronal death. The present study found the following: (1) PCA treatment reduced seizure-induced neuronal degeneration in the hippocampus; (2) PCA treatment reduced seizure-induced oxidative injury in the hippocampal neurons; (3) PCA treatment reversed seizure-induced GSH depletion in the hippocampal neurons; (4) PCA treatment reduced seizure-induced microglia activation.

Taken together, the present study suggests that PCA has an antioxidative effect and thus has therapeutic potential for the reduction of seizure-induced neuronal damage.

## Supplementary Materials

Supplementary materials can be found at www.mdpi.com/1422-0067/19/1/187/s1.
