# Supplementary material for: Protective Effects of Protocatechuic Acid on Seizure-Induced Neuronal Death
Source: Int J Mol Sci. 2018 Jan 8;19(1):187. doi: 10.3390/ijms19010187 (PMC5796136; doi:10.3390/ijms19010187)
Supplement: Supplementary file 1 [file ijms-19-00187-s001.pdf]

International Journal of
Molecular Sciences

MDPI

Protective Effects of Protocatechuic Acid on Seizure-Induced Neuronal Death

Song Hee Lee, Bo Young Choi, A Ra Kho, Jeong Hyun Jeong, Dae Ki Hong, Sang Hwon Lee, Sang Yup Lee, Min Woo Lee, Hong Ki Song, Hui Chul Choi and Sang Won Suh

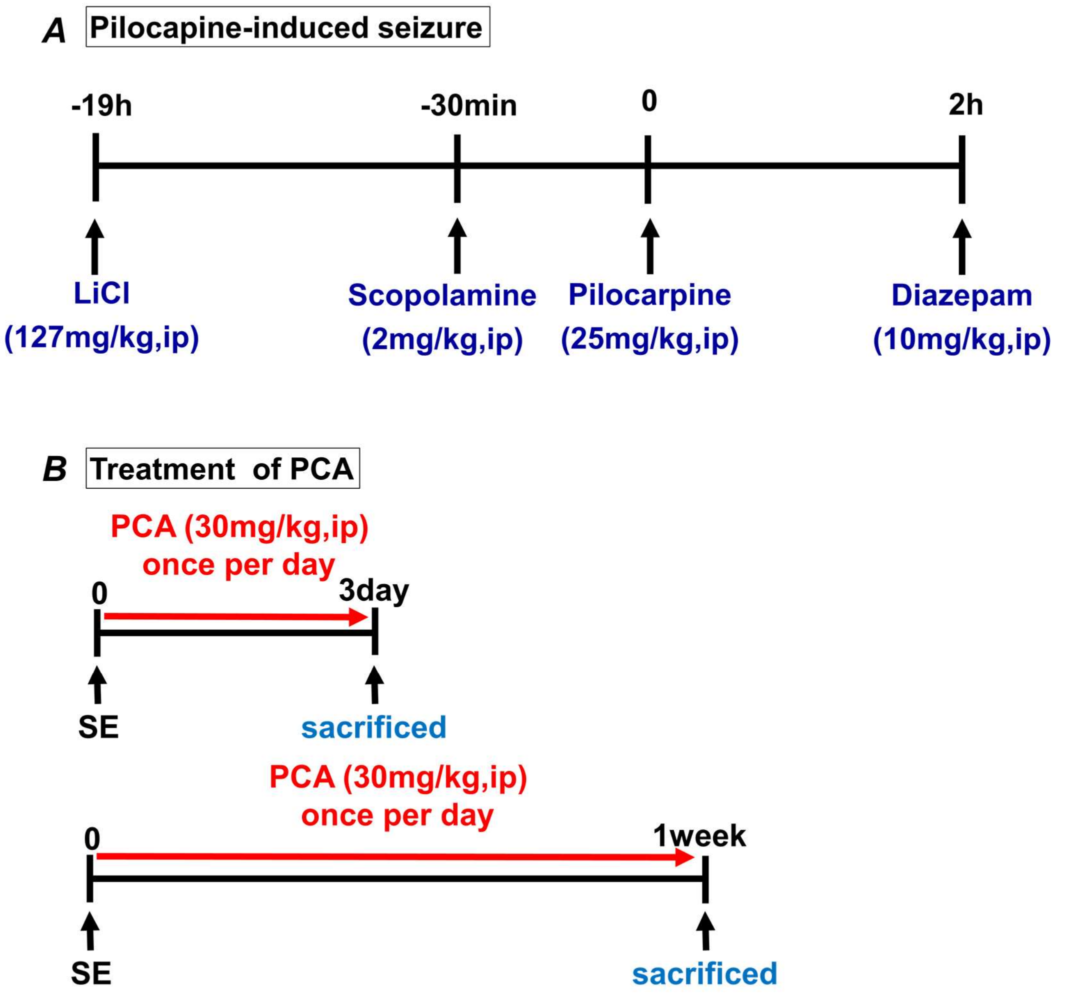

Figure S1. Supplementary figure shows the experimental procedure. (A) Experimental procedure in the Pilocarpine-induced seizure rats. (B) Experimental procedure for the duration of PCA administration.
